# Screening for oncogenic AF1q expression predicts disease recurrence in gastric cancer patients

**DOI:** 10.1038/s41598-024-67058-x

**Published:** 2024-07-10

**Authors:** Elisabeth S. Gruber, Georg Oberhuber, Michaela Schlederer, Peter Birner, Gerd Jomrich, Sebastian F. Schoppmann, William Tse, Lukas Kenner

**Affiliations:** 1 Division of Visceral Surgery, Department of General Surgery, Medical University Vienna, Waehringer Guertel 18-20, 1090 Vienna, Vienna Austria; 2 Department of Experimental and Animal Pathology, Clinical Institute of Pathology, Medical University Vienna, Waehringer Guertel 18-20, 1090 Vienna, Vienna Austria; 3 PIZ - patho im zentrum GmbH, St. Poelten, Lower Austria Austria; 4 Department of Medicine, School of Medicine, Case Western Reserve University, Cleveland, OH USA; 5 Immune Oncology Program, Case Comprehensive Cancer Center, Case Western Reserve University, Cleveland, OH USA; 6 Unit of Laboratory Animal Pathology, University of Veterinary Medicine Vienna, Vienna, Austria; 7 Christian Doppler Laboratory for Applied Metabolomics, Medical University Vienna, Vienna, Austria; 8 Center for Biomarker Research in Medicine (CBmed), Graz, Styria Austria

**Keywords:** AF1q, CD44, Lymph node stage, Recurrence, Survival, Gastric cancer, Gastric cancer, Surgical oncology

## Abstract

AF1q associates with tumor progression and metastases upon WNT signaling. The downstream WNT target CD44 has demonstrated prognostic significance in gastric cancer (GC). This study evaluates the impact of AF1q on tumor stage and survival in GC patients. Immunohistochemical marker expression was analyzed and data were processed to correlation and survival analysis. Out of 182 GC samples, 178 (97.8%) showed moderate to high AF1q expression (*p* < 0.001), these samples correlated with positive lymph node stage (*p* = 0.036). In a subgroup analysis of patients with nodal-positive GC (n = 129, 70.9%), enhanced tumoral AF1q expression resulted in impaired recurrence-free survival (RFS, *p* = 0.030). Enhanced tumoral CD44 expression resulted in impaired disease-specific survival (DSS) in the subgroup of patients with nodal-positive GC (*p* = 0.031) as well as in the overall GC group (*p* = 0.005). AF1q demonstrated as an independent prognostic marker for RFS (*p* = 0.035) and CD44 for DSS (*p* = 0.036). AF1q has shown potential for prognostication of RFS in GC patients and is predominantly expressed in nodal-positive GC. Testing AF1q provides a possibility of identifying patients with locoregional (and advanced) disease, particularly at risk for disease recurrence. Implementing AF1q into the diagnostic process may facilitate screening, prognosis estimation as well as consideration of preoperative multimodal treatment in patients qualifying for elective upfront surgery.

## Introduction

Gastric cancer (GC) represents a formidable global health challenge affecting both healthcare systems and the broader socio-economic landscape. The 5-year survival rate varies widely, depending on the stage at diagnosis and access to healthcare. However, GC lacks early symptoms and as screening is exclusively reserved for hereditary cancer syndromes, it is mostly diagnosed at an advanced stage. The tumors’ aggressive nature is accountable for early recurrence and poor outcome even in the stage of resectable, potentially curative disease^1^. Worldwide, the risk of dying from GC before the age of 75 is 10.7% and by the year 2040, the estimated number of deaths will rise from the recent 769,000 to 1.31 Mio. worldwide^2,3^. For this reason, multimodal treatment and consistent follow-up are critical components of addressing GC and alleviating its socio-economic impact^1^.

The molecular aspects of GC involve a multitude of genetic alterations and signaling pathways that drive disease initiation as well as progression. The oncogene *AF1Q*, also known as *MLLT11* (*Myeloid/Lymphoid or Mixed-Lineage Leukemia Translocated to 11*), was initially identified in hematological malignancies^4–6^. Beyond this, emerging evidence has highlighted its relevance in solid cancers, where the multifaceted oncoprotein AF1q modulates various cellular processes, from initiation and proliferation to dissemination and chemoresistance^7–11^. Recently, we have demonstrated that esophageal cancer patients with AF1q-positive tumors relapse and die earlier; in this previous study, we demonstrated a positive correlation with the potentially therapeutic WNT and STAT3 target genes CD44 and pYSTAT3 that may be transcriptionally co-activated by AF1q^12^. CD44 has proven to have prognostic and therapeutic potential in several cancer studies^13–17^ and since CD44 is known to be highly expressed in GC^18^, we here aimed to explore the role of AF1q in GC’s molecular landscape and clinical implications arising that might elicit promising avenues for screening, prognosis estimation as well as therapy.

## Results

### Patient and tumor characteristics

In total, 182 patients were included in the study (102 males, 80 females). Mean age was 69.0 years (34–92 years) and 149 patients were 69 years or older at time of diagnosis. Neoadjuvant chemotherapy was not administered routinely before 2011 and hence was not analyzed. Histopathological work-up of the resected tumor samples showed nodal-positive disease in 129 (70.9%) patients, 29 (15.9%) patients had distant metastatic disease; tumor grade showed differentiation as follows: good in 3 (1.6%), moderate in 50 (27.5%) and poor differentiation in 129 (70.9%) of patients; positive resection margin (R1) was found in 27 (14.8%) of the tumor samples. Macroscopically, 84 (46.2%) tumors were located in the corpus, 98 (53.8%) in the antrum.

### AF1q expression

**Figure 1:**
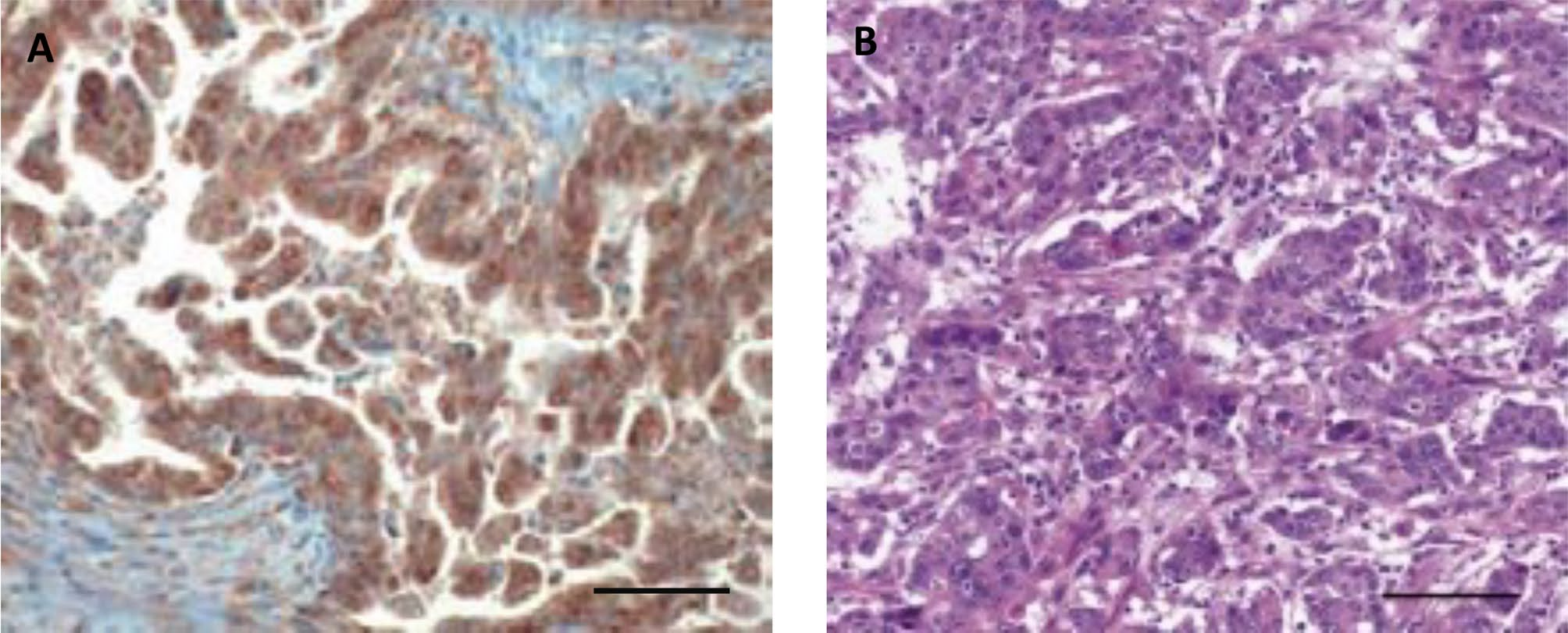
Representative examples of immunohistochemical (IHC) staining of AF1q in gastric adenocarcinoma. Samples with (**A**) enhanced AF1q expression versus samples with (**B**) hematoxylin–eosin staining of patients that underwent surgery. Scale bar 100 μm.

**Figure 2:**
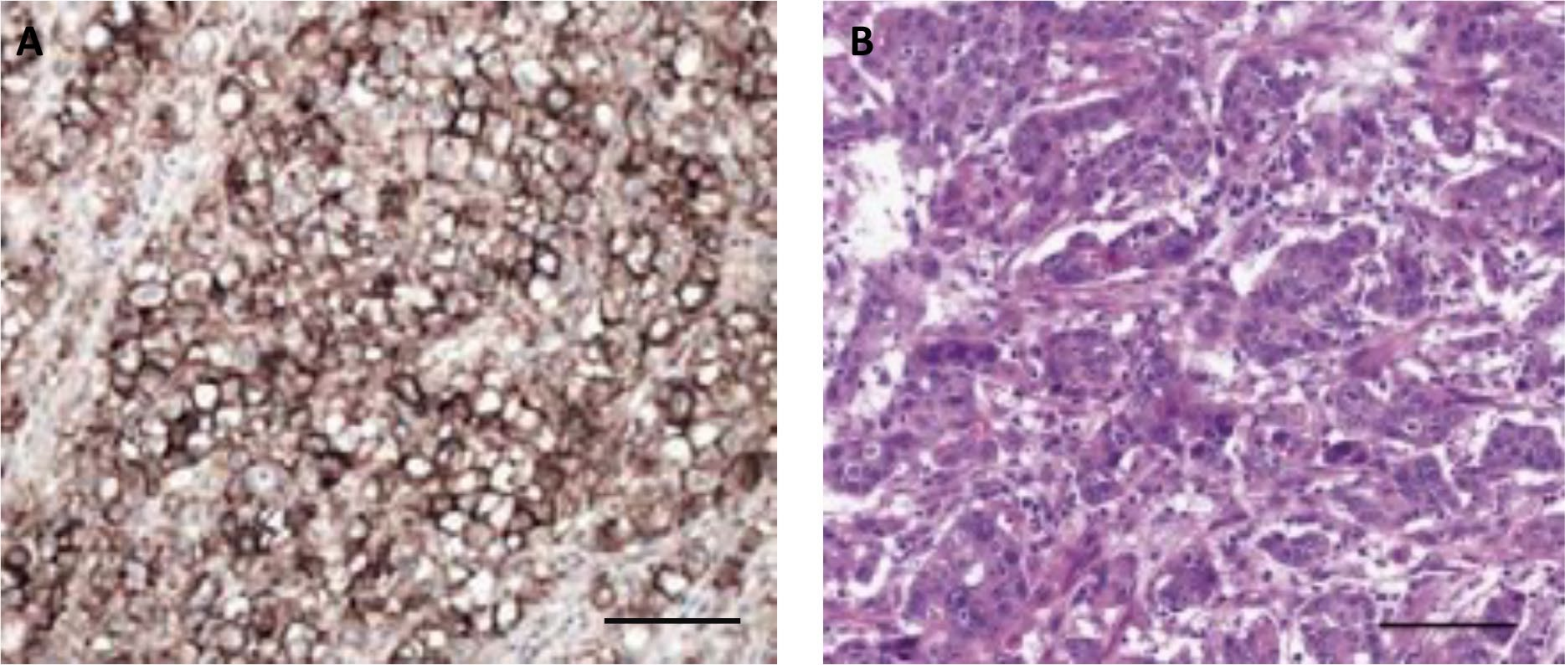
Representative examples of immunohistochemical (IHC) staining of CD44 in gastric adenocarcinoma. Samples with (**A**) enhanced CD44 expression versus samples with (**B**) hematoxylin–eosin staining of patients that underwent surgery. Scale bar 100 μm.

AF1q expression was found in 178 (97.8%) tumor samples (*p* < 0.001) and CD44 expression in 64 (35.2%) tumor samples (*p* = n.s.); 63 (35.4%) samples showed both AF1q and CD44 expression (*p* = n.s.). Whilst AF1q was predominantly expressed in nodal-positive GC (*p* = 0.036), CD44 expression showed enhanced expression in metastatic GC (*p* = 0.023). Immunohistochemical staining of AF1q and CD44 is shown in Figs. 1 and 2. AF1q expression in relation to patient and tumor characteristics is demonstrated in Table 1.

**Table 1:** AF1q expression in relation to patient and tumor characteristics.

Factor	Overall	AF1 positive	AF1 negative	*p*-value
Patient cohort	182 (100%)	178 (97.8%)	4 (2.2%)	**< 0.001**
CD44				n.s
Positive	64 (35.2%)	63 (35.4%)	1 (25%)	
Negative	118 (64.8%)	115 (64.6%)	3 (75%)	
Sex				n.s
Male	102 (56.0%)	100 (56.2%)	2 (50.0%)	
Female	80 (44.0%)	78 (43.8%)	2 (50.0%)	
Age (range)	69.0 (34–92)			n.s
< 69 years	88 (48.4%)	85 (47.8%)	3 (75%)	
≥ 69 years	94 (51.6%)	93 (52.2%)	1 (25%)	
UICC/AJCC				
pN+	129 (70.9%)	125 (96.9%)	4 (3.1%)	**0.036**
pM+	29 (15.9%)	29 (100.0%)	0 (0.0%)	n.s
R1	27 (14.8%)	26 (96.3%)	1 (3.7%)	n.s
Tumor grade				n.s
Good	3 (1.6%)	3 (1.6%)	0 (0.0%)	
Moderate	50 (27.5%)	48 (27.0%)	2 (50.0%)	
Poor	129 (70.9%)	127 (71.4%)	2 (50.0%)	
Recurrent disease	115 (63.2%)	111 (96.5%)	4. (3.5%)	n.s
Local	33 (18.5%)	33 (18.5%)	0 (0.0%)	
Distant	82 (81.5%)	78 (78.0%)	4 (3.5%)	
Disease-specific death	108 (59.3%)			n.s
Yes		104 (57.1%)	4 (100.0%)	
No		74 (40.7%)	0 (0.0%)	

Continuous variables are shown as median and range, categorical variables are expressed as absolute and relative numbers, n (%); pN and pM according to the AJCC/UICC staging system.

G: tumor grade; R: resection margin.

Significant values are in bold.

### Survival analysis

Median follow-up was 71 months (52–90 months) demonstrated recurrent disease in 115 (63.2%) patients and of those 33 (18.1%) had local recurrence. Out of 148 (81.3%) patients who died during the follow-up period, 108 (59.3%) patients died due to disease-specific reasons. Median survival times (and ranges) were calculated as follows: recurrence-free survival (RFS) 23.5 months (18–29 months), disease-specific survival (DSS) 43.7 months (34–54 months).

**Figure 3:**
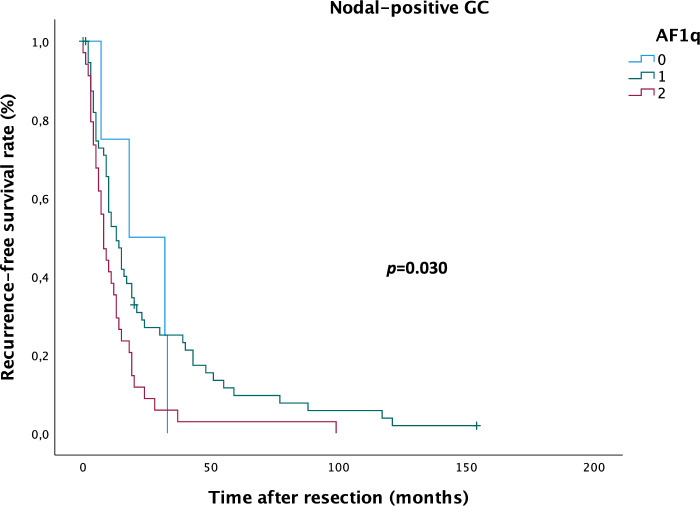
Kaplan–Meier analysis for recurrence-free survival in nodal-positive gastric cancer (GC) patients. Patients with moderate (1) and high (2) AF1q expression relapse earlier compared to patients with AF1q-negative (0) GC (log rank: *p* = 0.030).

In patients with AF1q-positive GC, median RFS was 26.8 months (19–34 months) in patients with moderate AF1q expression and 18.2 months (10–26 months) in patients with high AF1q expression compared to 22.5 (10–35 months) in patients with AF1q-negative GC; median DSS was 46.1 months (34–58 months) in patients with moderate AF1q expression and 37.0 months (22–52 months) in patients with high AF1q expression compared to 37.3 months (16–58 months) in patients with AF1q-negative GC. In the subgroup of nodal-positive GC, median RFS was 26.1 months (17–35 months) in patients with moderate AF1q expression and 13.1 months (7–19 months) in patients with high AF1q expression compared to 22.5 months (10–35 months) in patients with AF1q-negative GC; median DSS was 39.4 months (27–52 months) in patients with moderate AF1q expression and 26.8 months (23–42 months) in patients with high AF1q expression compared to 37.3 months (16–58 months) in patients with AF1q negative-GC. Enhanced tumoral AF1q expression resulted in significantly impaired RFS (Kaplan Meier/log rank; *p* = 0.030; Fig. 3), but not DSS in the subgroup of patients with nodal-positive GC. No significant impact was found on RFS or DSS in the overall GC group.

**Figure 4:**
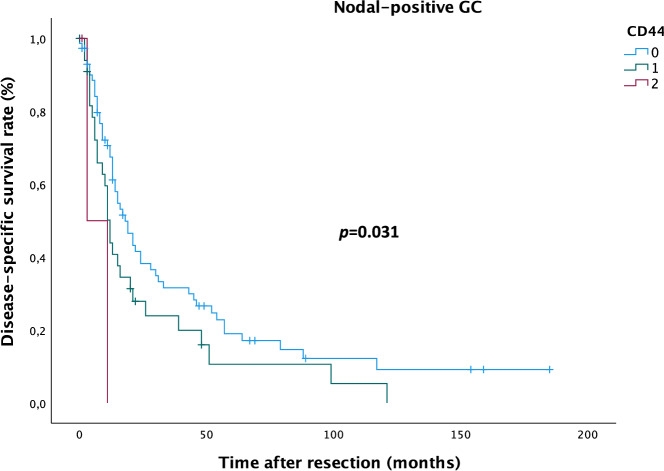
Kaplan–Meier analysis for disease-specific survival in nodal-positive gastric cancer (GC) patients. Patients with moderate (1) and high (2) CD44 expression die earlier compared to patients with CD44-negative (0) GC (log rank: *p* = 0.031).

**Figure 5:**
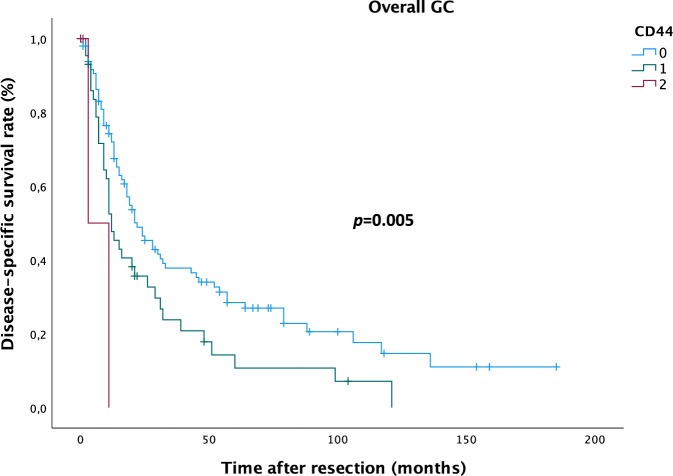
Kaplan–Meier analysis for disease-specific survival in gastric cancer (GC) patients. Patients with moderate (1) and high (2) CD44 expression die earlier compared to patients with CD44-negative (0) GC (log rank: *p* = 0.005).

In patients with CD44 positive GC, median RFS was 21.2 months (13–30 months) in patients with moderate CD44 expression and 6.0 months (0–12 months) in patients with high CD44 expression compared to 25.3 months (18–32 months) in patients with CD44 negative GC; median DSS was 28.1 months (17–39 months) in patients with moderate AF1q expression and 7.0 months (0–15 months) in patients with high AF1q expression compared to 51.8 months (38–65 months) in patients with AF1q negative GC. In the subgroup of nodal-positive GC, median RFS was 20.4 months (10–31 months) in patients with moderate CD44 expression and 6.0 months (0–12 months) in patients with high CD44 expression compared to 22.1 months (15–29 months) in patients with CD44 negative GC; median DSS was 25.6 months (13–38 months) in patients with moderate CD44 expression and 7.0 months (0–15 months) in patients with high CD44 expression compared to 41.4 months (28–55 months) in patients with CD44 negative GC. Enhanced tumoral CD44 expression resulted in significantly impaired DSS in the subgroup of patients with nodal-positive GC (DSS: Kaplan Meier/log rank; *p* = 0.031; Fig. 4) as well as in the overall GC group (DSS: Kaplan Meier/log rank; *p* = 0.005; Fig. 5), but no impact was found on RFS.

### Cox regression analysis

Patients with AF1q positive RC showed a trend towards a higher risk for disease recurrence (*p* = 0.071), but no impact on disease-specific death. Further prognostic factors for RFS and DSS are depicted in Table 2. In a multivariable Cox Regression model, AF1q proved to be an independent factor for RFS (*p* = 0.035) next to prognostic factors like lymph node stage, distant metastases, tumor grade and resection margin. With regard to DSS, AF1q showed a potential trend as an independent prognostic factor (*p* = 0.057) next to prognostic factors like lymph node stage, distant metastases, tumor grade (trend) and resection margin. Univariate and multivariable Cox Regression data are compiled in Table 2.

**Table 2:** Uni- and multivariate cox regression analysis for recurrence-free and disease-specific survival in GC patients.

Factor	Univariate *p*-value	Multivariable *p*-value	Hazard ratio	95% confidence interval	
				Low	High
Recurrence-free survival					
AF1q	0.071	**0.035**	1.5	1.0	2.2
CD44	**0.039**	n.s	n.a	n.a	n.a
pN	**0.006**	**0.043**	1.2	1.0	1.4
pM	**0.001**	**0.004**	2.1	1.3	3.5
G	n.s	**0.04**	1.6	1.0	2.6
R	**0.049**	**0.017**	1.9	1.1	3.1
Disease-specific survival					
AF1q	n.s	0.057	1.5	1.0	2.2
CD44	**< 0.001**	**0.036**	1.4	1.0	1.8
pN	**< 0.001**	**0.002**	1.4	1.1	1.6
pM	**< 0.001**	**< 0.001**	2.6	1.5	4.4
G	0.056	**0.016**	1.8	1.1	2.8
R	**< 0.001**	**< 0.001**	2.6	1.6	4.4

pN and pM according to the AJCC/UICC staging system.

G: tumor grade; R: resection margin.

Significant values are in bold.

## Discussion

Gastric cancer (GC) is the fifth most common cancer and the fourth common cause of cancer death worldwide, responsible for a total of 10 million deaths in 2020^3^. Though Out of those patients treated with curative intent, a considerable number relapse within two years after surgery (Asia: 60% vs. Europe: 36.8%)^19,20^. Exploring predictive markers to identify risk of recurrence is key to optimize follow-up and improve survival of GC patients. Whilst the role of the multifaceted oncogene *AF1Q* in GC is widely unknown, CD44 has proven prognostic potential for both diagnosis and treatment of GC^18^. We here focused on exploring the expression of AF1q in samples of patients operated on for GC as well as AF1q’s relation to tumoral CD44 expression; secondly, we aimed to elicit AF1q’s potential as a prognostic marker for GC survival. By analyzing 182 GC samples, we found AF1q to be significantly enhanced, especially in nodal-positive GC. In this subgroup of patients, those with AF1q-positive GC relapsed earlier, whilst those with CD44-positive tumors died earlier from their disease compared to marker-negative GC. Survival analysis revealed AF1q as an independent prognostic marker for RFS and solidifies the role of CD44 as an independent prognostic marker for DSS in GC patients.

In this GC cohort, 153 patients (84.1%) qualified for upfront surgery with postoperative chemotherapy, 115 patients (63.2%) suffered from disease recurrence and from these, 111 patients (96.5%) had AF1q-positive GC. Studies in human breast cancer samples demonstrated that the cooperation of AF1q with TCF7 is involved in the transcription of CD44^9^, a WNT target gene that is highly expressed in GC^18^ and known to drive tumor progression and epithelial-to-mesenchymal transition^21,22^, the basis for enhanced migratory capacity of cells and hence tumor spread. Additionally, other groups reported that AF1q associated with metastatic spread in colorectal, breast and lung cancer^23–26^. Although we had expected a possible association of AF1q with CD44 in GC since our recent study in esophageal cancer patients^12^, no association was found in the samples of this GC patient cohort, where AF1q was abundantly expressed compared to CD44 (AF1q: n = 178, 97.8% vs. CD44: n = 64, 35.2%), which is likely the reason for the lacking correlation between these markers. However, this association in terms of AF1q driving CD44 transcription would have been the assumed explanation that AF1q associates with nodal-positive and recurrent GC and since this rather low CD44 expression in GC is contradictive to the literature, the task of how AF1q is involved in tumor spread mechanistically now still remains open for further analysis. Nonetheless, patients with CD44-positive GC in this cohort died earlier from their disease, which underlines the potency of CD44 as a tumor modulator—given the fact that downregulation of CD44 inhibits proliferation, invasion and metastasis of GC^13,15,17^, rendering it a potential therapeutic target for GC.

Until the year 2040, Asia will account for the highest number of GC-related deaths (Asia 1.01 million vs. Europe 124,000^2^; geographical variations expected due to *Helicobacter pylori* infection, smoking and consumption of salt and salt-preserved foods^27–29^). Especially in GC, where screening is not recommended routinely, assessment of appropriate marker expression might facilitate the diagnostic process and optimize treatment decisions as well as follow-up. Patients with AF1q-positive GC showed significantly impaired RFS (Fig. 3) and as for this, including marker analysis into the diagnostic process might prompt multimodal treatment in terms of escalating to preoperative therapy in selected cases of *de-facto* resectable GC; this “pseudo-neoadjuvant” regimen would aim to combat locoregional disease up to dormant metastases and ultimately prevent disease recurrence and consequently death from disease. Patients with CD44-positive GC showed significantly impaired DSS in the subgroup of patients with nodal-positive GC (Fig. 4) as well as in the overall GC group (Fig. 5), but no impact was found on RFS. This finding and the correlation with metastatic GC raises the assumption that CD44 might operate through a more aggressive tumor behavior, which results in earlier death from disease.

In conclusion, this study provides evidence that AF1q has a potential as a negative and independent prognostic marker for RFS in patients with GC. The expression especially in patients with nodal-positive GC justifies considering including AF1q into the diagnostic process and through this to prompt preoperative multimodal treatment even in upfront resectable GC to limit locoregional as well as recurrent disease. This treatment regimen would be a step towards earlier diagnosis, better prognosis estimation and lower socio-economic burden of this still fatal disease.

## Methods

### Patient cohort

Patients operated on for gastric adenocarcinoma between 1992 and 2011 at the Medical University of Vienna were included in the study. Ethical approval for the study was obtained from the institutional review board (‘Ethikkommission’ of the Medical University of Vienna, protocol #1197/2019) and informed consent was waived off. All methods were carried out in accordance with relevant guidelines and regulations.

Histopathological staging was conducted according to the AJCC/UICC staging system^1^. Surgical specimens were carefully selected by an experienced, board-certified pathologist. Selected tumor samples were represented by triplicate core biopsies to construct a tissue microarray (TMA), which were then cut and processed to immunohistochemical staining.

### Immunohistochemistry

The expression of AF1q as well as CD44 was evaluated in resected human GC samples. Immunostaining was performed using a standard protocol with the following antibodies: AF1q (Abcam, ab109016; 1:200), CD44 (Santa Cruz, sc-9960; 1:200). Paraffin sections were de-waxed, and for the antigen retrieval, a citrate buffer pH 6 (CD44) or a Tris/Ethylenediaminetetraacetic acid (EDTA) buffer pH 9 (AF1q) was used. After endogenous peroxidase blocking, avidin and biotin blocking steps were performed. The antibodies were incubated overnight at 4 °C in PBS + 1% bovine serum albumin (BSA). Slides were washed with phosphate buffered saline (PBS) the following day and incubated with polyvalent-secondary antibody (IDetect Super Stain System HRP, ID laboratories) and horseradish peroxidase (HRP; IDetect Super Stain System HRP, ID laboratories). Signals were visualized with 3-amino-9-ethylcarbazole (ID laboratories). After counterstaining with hemalaun, the slides were mounted. The samples were analyzed by an experienced, board-certified pathologist. A specimen was considered as positive when at least 50% of tumor cells showed moderate or strong cytoplasmic (AF1q) or membranous (CD44) marker expression. Antibody specificity has been confirmed in previous studies^9,12^.

### Statistical analysis

To evaluate AF1q expression in relation to patient and tumor characteristics, the Chi^2^ test and the Spearman rank correlation coefficient (r_s_) were used as appropriate. The Kaplan–Meier method was used to estimate survival using the log-rank test for group comparisons. Survival times were defined as follows: RFS—time from surgery until disease recurrence, DSS—time from surgery until death from disease. To evaluate the prognostic potential of AF1q, a multivariable Cox Regression model was calculated including AJCC/UICC tumor staging and resection margin^1,30^. For statistical computing IBM^®^ SPSS^®^ Statistics Version 28.0.0.0 (IBM Cooperation, USA) was used. For all analyses, a two-sided *p*-value less than 0.05 was considered statistically significant.

## Data Availability

To protect patient privacy, the data related to patients cannot be available for public access. However, they can be obtained from the corresponding authors on reasonable request approved by the institutional review board.
